# Application of fungal fluorescent staining in oral candidiasis: diagnostic analysis of 228 specimens

**DOI:** 10.1186/s12866-019-1467-x

**Published:** 2019-05-14

**Authors:** Yilin Yao, Linjun Shi, Chunye Zhang, Hong Sun, Lan Wu

**Affiliations:** 1 Department of Oral Mucosal Diseases, Shanghai Ninth People’s Hospital, College of Stomatology, Shanghai Jiao Tong University School of Medicine, Shanghai, China; 2 National Clinical Research Center for Oral Diseases, Shanghai, China; 3 Shanghai Key Laboratory of Stomatology & Shanghai Research Institute of Stomatology, Shanghai, China; 4 Department of Oral Pathology, Shanghai Ninth People’s Hospital, College of Stomatology, Shanghai Jiao Tong University School of Medicine, Shanghai, China; 5 Department of Laboratory Medicine, Shanghai Ninth People’s Hospital, Shanghai Jiao Tong University School of Medicine, Shanghai, China

**Keywords:** Fungal fluorescent staining, Oral candidiasis, Fungal culture, Periodic acid-Schiff reagent staining

## Abstract

**Background:**

Several conventional methods, including fungal culture and periodic acid-Schiff (PAS) reagent staining, have been used to diagnose oral candidiasis. The aim of this study was to evaluate the efficacy of a novel method, fungal fluorescent staining, in relation to conventional protocols in the diagnosis of oral candidiasis.

**Methods:**

We collected 106 oral swabs and 122 oral biopsy tissues from patients highly suspected with oral candidiasis. We applied fungal culture and periodic acid-Schiff reagent staining as the gold standard diagnostic tools. The efficacy of these methods in determining the presence of *Candida* was compared with that of fluorescent staining.

**Results:**

In the majority of specimens subjected to fluorescent staining, fungal organisms were distinguished by blue fluorescence surrounding their tubular or annular shapes. The sensitivity, specificity, Youden index, positive predictive value and negative predictive value of the fluorescent staining method were 82.7, 93.5, 76.7, 96.8 and 69.1% in oral swabs and 90.0, 92.9, 82.9, 96.0 and 82.9% in oral biopsy tissues, respectively.

**Conclusions:**

Fungal fluorescent staining represents a rapid method for detection of *Candida*, supporting its potential utility as an effective early diagnostic tool for oral candidiasis.

## Background

*Candida*, an opportunistic pathogen, is a commensal and harmless organism under normal conditions that becomes invasive; pathogenic pseudohyphae overbreed locally and cause oral candidiasis upon disruption of the balance of flora or debilitationof the host [1, 2]. The disease is most commonly caused by overgrowth of *Candida albicans* in the mouth [3]. Diagnosis of oral candidiasis is generally based on thorough analysis of medical history and physical examination, and confirmed by examining oral swabs or oral biopsy tissue samples. The conventional methods adopted for diagnosis in these two sample types are fungal culture [4–6] and PAS staining [4, 7, 8]. However, both techniques are time-consuming and lead to false-negative results in 5–15% cases [9], highlighting the necessity for more rapid and sensitive methods. Fungal fluorescent staining is currently under investigation as a potential means to effectively detect *Candida* infection. While several studies have documented the successful application of fluorescent staining for fungal detection in nail specimens [10–12], limited reports are available on diagnosis using oral samples. This study was designed to compare fungal fluorescent staining with conventional methods, with a view to establishing an optimal technique for accurate and rapid diagnosis of oral candidiasis.

## Results

### Clinical specimens and participants

**Fig. 1:**
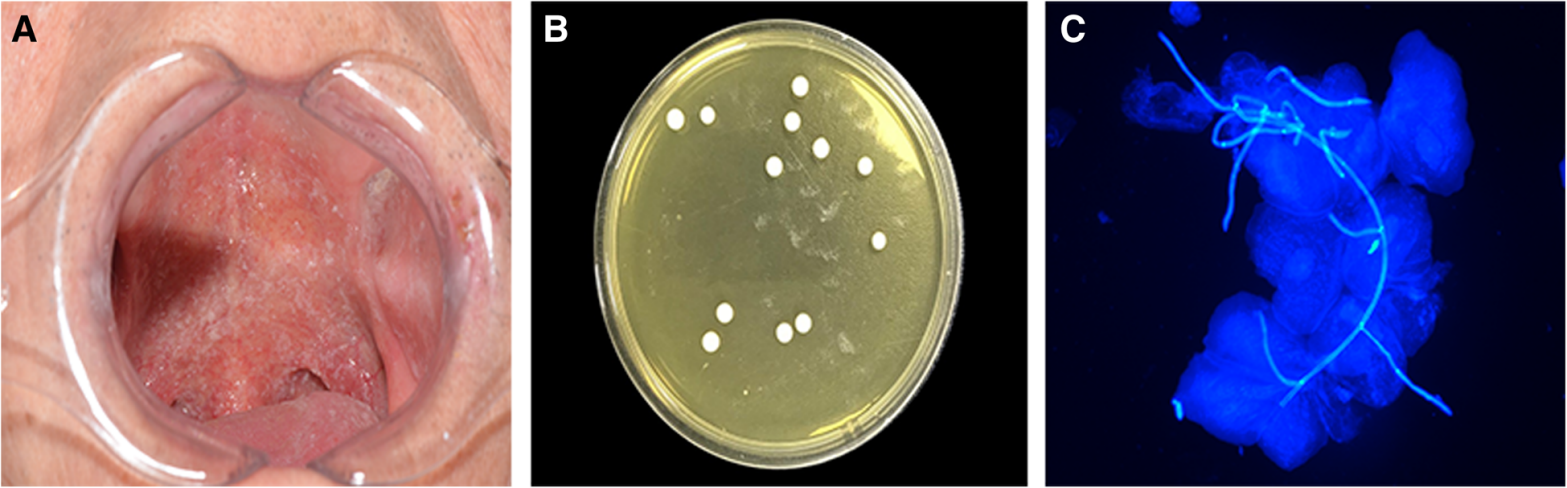
**a** Clinical manifestation of highly suspected oral candidiasis. **b** Presence of *Candida* colonies in the culture-positive case. **c** Fungal hyphae surrounded by strong blue fluorescence under fluorescence microscopy (× 40)

**Fig. 2:**
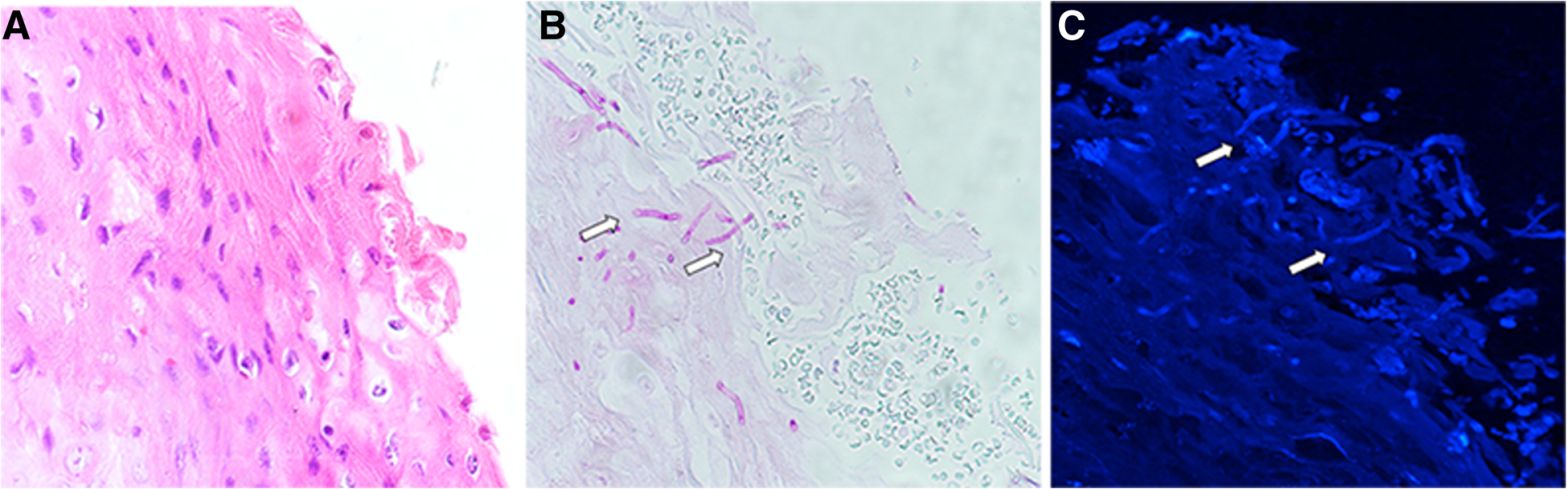
**a** A case of highly suspected oral candidiasis via HE staining under light microscopy (× 40). **b** Fungal organism (white arrows) on PAS of same specimen (×40). **c** Fungal organisms from the same section as B distinguishable via fluorescence microscopy as tubular or tubulo-annular structures (white arrows) with a thin rim of bright blue fluorescence (× 40)

The features of 228 specimens with suspected oral candidiasis (Figs. 1a and 2a) are summarized in Table 1. In the first phase, 106 oral swabs were obtained from 41 males and 65 females aged 25–78 years (average age, 47.6 years). Overall, 66 males and 56 females participated in the second phase of the study. Participant ages ranged from 27 to 82 years (median age, 55.9 years). The majority of biopsy tissues were histologically diagnosed as oral leukoplakia (59.8%), followed by oral lichen planus (15.6%), oral squamous cell carcinoma (14.8%) and oral mucositis (9.3%).

**Table 1:** Baseline characteristics of 228 specimens

Feature	Swabs	Biopsy tissues
Total	106	122
Age(years)		
Mean	47.6	55.9
Range	25–78	27–82
Sex		
Males	41(38.7%)	66(45.9%)
Females	65(61.3%)	56(54.1%)
Histology		
Oral squamous cell carcinoma	/	18(14.8%)
Oral leukoplakia	/	73(59.8%)
Oral lichen planus	/	19(15.6%)
Oral mucositis	/	12(9.8%)

### Fungal detection in oral swabs

In culture-positive cases, unique fungal colonies of *Candida* (Fig. 1b) were found on Sabouraud’s Dextrose Agar (SDA) that were white to cream in color, smooth, glabrous, with a yeast-like appearance and smell. Upon growth on Chromogenic Candida Agar (CCA), colonies of *C. albicans* are characteristically green, *C. glabrata* purple, and *C. tropicalis* bluish-grey.

In fluorescence-positive cases, hyphae (Fig. 1c) and spores appeared bright blue against a black background, and fungal morphology was easily distinguishable in oral swabs.

Among the 106 oral swabs, fungal culture was positive in 75 samples (70.7%), among which 13 turned out negative with the fluorescent staining technique, and negative in 31 samples (29.3%), among which 2 were positive with fluorescent staining. These data are presented in Table 2. Among the culture-positive samples, *C. albicans* was detected in 58 cases, *C. glabrata* in 10 cases and *C. tropicalis* in 7 cases. Importantly, the fluorescent staining technique required an average time of only 10 min while fungal culture was conducted over 6 days.

**Table 2:** The results of *Candida* detection in oral swabs

Technique	Culture		Total
	Positive	Negative	
FLO			
Positive	62	2	64
Negative	13	29	42
Total	75	31	106

*FLO* fluorescent staining

### Fungal detection in oral tissues

In PAS-positive cases, *Candida* hyphae invaded the epithelium at right angles to the surface (Fig. 2b). The fungal appearance of fluorescent staining in tissue specimens was similar to that in oral swabs but not as easy to distinguish (Fig. 2c).

In total, 80 specimens (65.6%) were PAS-positive, among which 8 displayed negative fluorescent staining, and 42 (34.4%) were PAS-negative, among which 3 were detected as positive using fluorescent staining. These data are presented in Table 3. The fluorescent technique required 1 min to generate specimens and 10–15 min to read samples, compared to a longer period of 2 h for specimen generation and 10–20 min for reading the results with PAS staining.

**Table 3:** The results of *Candida* detection in oral biopsy tissues

Technique	PAS		Total
	Positive	Negative	
FLO			
Positive	72	3	75
Negative	8	39	47
Total	80	42	122

*FLO* fluorescent staining, *PAS* periodic acid-Schiff reagent staining

### Sensitivity, specificity, Youden index, and negative and positive predictive values

For determination of each parameter, conventional methods (culture−/PAS-positive) were used as the gold standard. For oral swabs and oral biopsy tissues, the sensitivity of fluorescent staining was 82.7 and 90.0%, and specificity was 93.5 and 92.9%, respectively. The positive predictive values were 96.8 and 96.0%, negative predictive values were 69.1 and 82.9%, and Youden indexes were 76.2 and 82.9%, respectively (> 70%). All three diagnostic methods for oral candidiasis are compared in Table 4.

**Table 4:** Sensitivity, specificity, positive and negative predictive value and Youden index for all diagnostic methods

Specimens	Oral swabs	Oral biopsy tissues
Sensitivity	82.7%	90.0%
Specificity	93.5%	92.9%
Positive predictive value	96.8%	96.0%
Negative predictive value	69.1%	82.9%
Youden index	76.7%	82.9%

## Discussion

The diagnosis of oral candidiasis is fundamentally clinical [1]. Microbiological techniques or culture are often used to identify hyphae or spores for confirmation of diagnosis [13]. Timely and efficient laboratory diagnosis is essential to facilitate the implementation of effective antifungal treatments. In this study, we evaluated the utility of fluorescent staining in the diagnosis of oral candidiasis, with a view to establishing a clinically appropriate rapid and efficient *Candida* detection technique. The efficacy of fungal fluorescent staining as a diagnostic tool was compared with conventional fungal culture and PAS staining methods.

Fungal fluorescent staining is a novel diagnostic method for oral candidiasis. The fluorescent antibody in the Calcofluor white (CFW) fluorescent dye specifically binds to glucan and chitin layers unique to the fungal cell wall [14]. Thus, fluorescein, which emit blue fluorescence under illumination, is indirectly labeled upon specific binding to the fungal cell wall. And then clear visualization of the profile of the fungus was observed. In a diagnostic study by Okamoto MR [5] using culture as the gold standard, fluorescent staining had 84% sensitivity and 100% specificity, resulting in a positive predictive value of 100% and negative predictive value of 65%. Kirani KR [15] conducted a similar investigation, which disclosed 100% sensitivity, 93.3% specificity, a negative predictive value of 100% and a positive predictive value of 85.7%. The sensitivity of fluorescent staining was high, supporting its value in the diagnosis of *Candida* infection. Using fungal culture as the gold standard in this study, the sensitivity, specificity, Youden index, positive predictive value and negative predictive value obtained with the fluorescent staining method were 82.7, 93.5, 76.7, 96.8 and 69.1%, respectively, further validating the suitability of this method. It is important to note that fluorescent staining requires only 10 min, compared with 6 days of fungal culture, further providing a guarantee of early diagnosis. Experienced staff found fluorescent staining preparations easy and rapid and the appearance of hyphae or spores bright and distinguishable using fluorescence microscopy [16]. Overall, the fluorescent technique was safe, quick and reliable, all of which are important considerations in a busy diagnostic laboratory.

One of the earliest descriptions of fungal fluorescence in tissue sections was reported by Graham in 1983 [17], who recommended fluorescent staining as a supplementary detection technique. Subsequently, Elston [18] reported the application of fluorescent staining in nail samples, 66% of which were positive. Additionally, moderate to strong fluorescence was detected in 74% oral squamous cell carcinoma tissues by Jahanshahi [19]. Data from both studies were consistent with our results, showing strong fluorescence in 61.5% of specimens. In this investigation, detection of fluorescence was related to the density of fungi in specimens and background fluorescence of keratin, which could be confused with fungal fluorescence. However, a similar problem exists with PAS staining [19]. Moreover, distinguishing species based on morphology is a challenge with both methods. Accurate identification of fungal species may be achieved in conjunction with other techniques, such as immunohistochemistry [20], in situ hybridization [21] and polymerase chain reaction [22]. In our tissue analysis, fungal fluorescent staining showed high sensitivity (90.0%), specificity (92.9%) and Youden index (82.9%), validating the conclusions of Graham [17]. Fluorescent staining of oral biopsy tissues has several distinct advantages to PAS staining. Firstly, results are obtained almost instantaneously. Secondly, this method is a more economical option than PAS. Thirdly, tissue sections that have been stained with fluorescence dyes can be reused.

Above all, our findings support the utility of fungal fluorescent staining in early diagnosis of oral candidiasis. We conclude that fluorescent staining serves as a rapid, simple and convenient screening tool for detection of fungi in oral specimens.

## Conclusions

Accuracy and speed are the highly desirable goals for fungal detection. The time required for fluorescent staining is shorter than that for the corresponding gold standard in oral swabs and oral biopsy tissues. In both specimen types, fungal fluorescent staining shows high sensitivity, specificity and Youden index supporting its value as a potential new method for early detection of *Candida*.

## Methods

### Clinical specimens

The study was divided into two phases. In the first phase, 106 patients with clinically suspected oral candidiasis were enrolled. Two oral swabs were obtained at the same time from lesions of the patients. Written informed consent based on the guidelines and agreements of the institutional ethical committee was obtained. All specimens were collected by the same experienced mycologist. In the second phase, 122 hematoxylin-eosin (HE)-stained tissue specimens of oral mucosal diseases with suspected *Candida* colonization between 2010 and 2018 were retrieved from the archive and two serial sections obtained from each specimen.

### Fungal detection in oral swabs

In the first phase, two oral swabs were examined via culture and fungal fluorescent staining. For fluorescent staining, the swab was applied onto a glass slide. A 50 μL drop of CFW fluorescent dye (Liming Biotech Co., Ltd., Nanjing, China) was placed on the slide for 30 s and excess dye gently removed with blotting paper. The slide was covered with a cover glass and examined under a fluorescence microscope (Nikon Ni-U, Japan) using blue light excitation (300–400 nm for emission wavelength with excitation at around 355 nm).

A sample culture was performed in parallel on SDA (Hope Biotech Co., Ltd., Qingdao, China) at 25 °C for 5–7 days. The rate and texture of growth, surface color, and color on reverse and diffusible pigments were determined. Culture-positive strains were subsequently streaked on CCA (Chromagar Microbiology, Paris, France) and further incubated at 37 °C for 24 h. Candida species were identified by colony color according to the manufacturer’s chart.

### Fungal detection in oral biopsy tissues

In the second phase, two sections were used for diagnosis via PAS and fungal fluorescent staining. In the fluorescent staining procedure, after dewaxing the sample,the same operation procedure was performed as before.

In terms of PAS staining, sections were incubated in 0.1% periodic acid for 5–8 min after dewaxing, washed in running tap water for 2–3 min and immersed in Schiff’sreagent for 10–20 min. Subsequently, sections were washed in tap water for 10 min, counterstained with hematoxylin for 2 min, differentiated in acidic ethanol for 2–3 s and washed in tap water for 10 min. Finally, sections were dehydrated with ethanol, cleared in xylene and mounted with neutral balsam (Sinopharm Chemical Reagent Co., Ltd., Shanghai, China).

Prior to the study, all participants were trained in the relevant Standard OperationProcedures (SOPs). Diagnosis of a sample was assessed by two experts in oral pathology or laboratory medicine who reached an inter-observer agreement. The total time taken for each experiment was recorded.

### Statistical analysis

Sensitivity, specificity, Youden index, and negative and positive predictive value were calculated using fungal culture as the gold standard for the first phase and PAS staining as the gold standard for the second phase.

## Abbreviations

CCA: Chromogenic Candida Agar
CFW: Calcofluor white
HE: Hematoxylin-eosin
PAS: Periodic acid-Schif
SDA: Sabouraud’s Dextrose Agar
